# Association between race/skin color and premature birth: a systematic review with meta-analysis

**DOI:** 10.11606/S1518-8787.2018052000406

**Published:** 2018-03-14

**Authors:** Kelly Albuquerque de Oliveira, Edna Maria de Araújo, Keyte Albuquerque de Oliveira, Cesar Augusto Casotti, Carlos Alberto Lima da Silva, Djanilson Barbosa dos Santos

**Affiliations:** I Universidade Estadual de Feira de Santana. Programa de Pós-Graduação em Saúde Coletiva. Feira de Santana, BA, Brasil; II Universidade Estadual de Feira de Santana. Departamento de Saúde. Feira de Santana, BA, Brasil; III Faculdade Nobre. Feira de Santana, BA, Brasil; IV Universidade Estadual do Sudoeste da Bahia. Departamento de Saúde. Jequié, BA, Brasil; V Universidade Federal do Recôncavo da Bahia. Centro de Ciências da Saúde. Santo Antônio de Jesus, BA, Brasil

**Keywords:** Infant, Premature, epidemiology, Ethnicity and Health, Meta-Analysis, Nascimento Prematuro, epidemiologia, Origem Étnica e Saúde, Metanálise

## Abstract

**OBJECTIVE:**

To analyze the association between race/skin color and the occurrence of prematurity.

**METHODS:**

Meta-analysis with observational studies, selected by a systematic review in the bibliographic databases Medline and Biblioteca Virtual da Saúde with the descriptors: “Race or ethnic group” and “ethnicity and health” associated with the words “infant premature” and “obstetric labor premature”. Articles published in the period from 2010 to 2014, of the observational epidemiological type, in Portuguese, English and Spanish, were included. Articles that did not have abstracts or that were review articles, theses, dissertations, and editorials were excluded. We adopted the relative risk and their respective confidence intervals (95%CI) as measures of effect, obtained through the random effect model and represented by the forest plot type graph. The Egger test and the Newcastle-Ottawa scale, respectively, were used to analyze possible publication biases and the quality of the studies.

**RESULTS:**

Of the 926 articles identified, 17 were eligible for the study. Of the 17 full texts published, seven were retrospective cohort studies, nine were cross-sectional studies, and one was a case-control study. Except for one study, the others reported a positive association between race/color of skin and prematurity. Compared with full-term newborns, the relative risk of the combined effect in those born preterm was 1.51 (95%CI 1.39-1.69). The funnel chart suggested publication bias.

**CONCLUSIONS:**

The present meta-analysis indicated a positive association for the risk of prematurity according to race/skin color.

## RESUMO

**OBJETIVO:**

Analisar a associação entre raça/cor da pele e a ocorrência da prematuridade.

**MÉTODOS:**

Meta-análise com estudos observacionais, selecionados por revisão sistemática em bases de dados bibliográficos Medline e Biblioteca Virtual da Saúde com os descritores: “*Race or ethnic group*” e “*ethnicity and health*” associados às palavras “*infant premature*” e “*obstetric labor premature*”. Foram incluídos os artigos publicados no período de 2010 a 2014, do tipo epidemiológico observacional, nas línguas portuguesa, inglesa e espanhola. Foram excluídos os artigos que não possuíam resumos ou que fossem artigos de revisão, teses, dissertações e editorias. Foi adotado o risco relativo e seus respectivos intervalos de confiança (IC95%) como medidas de efeito, obtidos por meio do modelo de efeito aleatório e representados a partir do gráfico do tipo *forest plot*. Para analisar os possíveis vieses de publicação e qualidade dos estudos, foi utilizado o teste de Egger e a escala de Newcastle-Ottawa, respectivamente.

**RESULTADOS:**

Dos 926 artigos identificados, 17 foram elegíveis para o estudo. Dos 17 textos completos publicados, sete eram estudos de coorte retrospectiva, nove eram transversais e um era caso-controle. Com exceção de um estudo, os demais relataram associação positiva entre a raça/cor da pele e a prematuridade. Comparado com recém-nascidos a termo, o risco relativo do efeito combinado naqueles que nasceram prematuros foi de 1,51 (IC95% 1,39–1,69). O gráfico de funil sugeriu viés de publicação.

**CONCLUSÕES:**

A presente meta-análise indicou uma associação positiva para o risco da prematuridade segundo a raça/cor da pele.

## INTRODUCTION

Prematurity (PMT) is an important indicator of child health since it increases the risk of chronic diseases^1^. It represents the largest cause of neonatal morbidity and mortality in the world and generates extremely high costs for countries^2^.

A systematic review of the incidence of prematurity in the world estimated that 12.9 million births were premature, about 9.6% of all births worldwide. Of these, 85.0% were concentrated in Africa and Asia, with 10.9 million premature births, 0.5 million in Europe and North America, and 0.9 million in Latin America and the Caribbean^3^.

The prevalence of preterm births in the United States increased from 9.5% in 1981 to 12.7% in 2005, and is currently in the range of 12.0% to 13.0%, while in Europe these values range from 5.0% to 9.0%^4^. In Brazil, it was possible to observe the temporal growth trend in the rates of prematurity. The SINASC data analysis showed that the prevalence was 5.0% in 1994, 5.4% in 1998, 5.6% in 2000, and 6.5% in 2004^5^; the prevalence was 6.0% and 7.0%, from 2000 to 2010, with Sinasc data. The values were between 11.0% and 12.0% for the same period after correction and 11.7% to 11.8% for the triennium 2009–2011^6^.

Socioeconomic and ethnic-racial inequality is documented as a risk for the occurrence of PMT^7^. Regarding ethnic-racial inequality, as, for example, in the United States, race/color has been evidenced as an important social determinant for the health of the population, since the treatment of the race/skin color variable only as genetic variation does not explain health differentials by different color groups^8–11^.

In this sense, it is important to elucidate the theory of social determination, in which the social position occupied by the individual contributes significantly to the occurrence of diseases and also to their unequal distribution^12–15^.

Thus, the race/color variable must be discussed as a social determinant in the occurrence of diseases and health problems and not as mere biological determinism, since race/color carries a social-historical framework in the group differentials.

Studies have shown that the incidence rates of premature births are unequal according to race/skin color. Black women are 2.5 times more likely to have preterm birth compared to white women, and these racial differences have increased since 1990^7^ ^,^ ^16^ ^,^ ^17^.

There are evidence that ethnic-racial disparities can lead to prematurity. A study conducted in the United States found considerable variation in birth outcomes by maternal race/skin color: A total of 18.4% deliveries by black women occurred before 37 weeks of gestation^18^. A cohort study in the United Kingdom with the objective of comparing the duration of gestation among white women and black women concluded that the latter are 1.5 times more likely to have preterm births^19^.

In Brazil, a persistent unfavorable situation was observed for black and brown women. They were less likely to undergo gynecological and prenatal consultations and have even fewer chances to perform the first prenatal visit in a period equal to or lower than the fourth month of pregnancy^6^ ^,^ ^20^. Data from a cohort of Ribeirão Preto, state of São Paulo, showed that skin color is an independent risk factor for PMT, even after adjustment of family income and maternal schooling. This suggests that racial differences in relation to PMT are explained by the socioeconomic disadvantage experienced by black women, but they are also influenced by other factors, possibly by racial discrimination^21^.

The analysis of the results from births due to ethnic or racial differences in mothers is necessary, since they may suggest etiological hypotheses, highlight the implications on gestational outcomes and direct to improve the quality of prenatal consultations^22^ ^,^ ^23^. Although several systematic reviews have been published on the association between some aspects of skin color/race in relation to PMT^7^ ^,^ ^16^, the authors of the present study identified a meta-analysis only relating the occurrence of PMT directly to race/skin color. They selected articles from 1983 to 2011 and worked with adjusted association measures^24^. Thus, in order to provide evidence and systematize information about the association between race/skin color and PMT, this study was conducted with the purpose of providing information to support the planning of future studies and public policies for the prevention of preterm birth and its consequences for the quality of life of the population and reduction of health services costs.

The hypothesis of this study is that black women are at a higher risk of having preterm deliveries when compared to non-black women. The objective of the present study was to perform a meta-analysis to analyze the association between the race/skin color of pregnant women and the occurrence of PMT.

## METHODS

Systematic review with meta-analysis, based on the Preferred Reporting Items for Systematic Reviews and Meta-Analyzes (PRISMA), which consists of 27 items that help in the elaboration, analysis, publication of meta-analyses and systematic reviews of observational studies^25^. A research protocol was developed to aid in the search, extraction of data, analysis, and interpretation of articles.

We included the articles with an epidemiological observational design that met the following criteria: original with abstract available, published in the last five years (2010 to 2014), published in Portuguese, English, and Spanish, and presented only primary data to calculate the measure of association between race/skin color and prematurity. Articles without abstracts and articles of review, theses/dissertations, and editorials were excluded.

For the article search, the integrated search system of the *Biblioteca Virtual em Saúde Brasil* (BVS), Medline and PubMed were used, which are databases that collect publications of scientific journals in health. We aimed to find publications of scientific articles related to the association between race/skin color and gestational prematurity.

The integrated search method was used for searches on all indexes and all sources. This allowed a wide search, integrating several databases and a detailed search, by the relevance of titles, abstracts, and texts. The words used for the search were: “Race or ethnic group” and “ethnicity and health” associated with the words “infant premature” and “obstetric labor premature”.

When doing the research using the association of descriptors, in the BVS of October and November 2015, 1,163 results of texts were found, of which 372 were texts published as of 2010. The search in Medline and PubMed using the same descriptor association found 1,907 results, 554 published as of 2010.

Participating in the study were pregnant women who recorded gestational age at the birth of the newborn. The outcome was PMT, referenced as the occurrence of the birth before the 37th week of gestation. The exposure variable was race/skin color. All participants of black race/skin color, which corresponded to black and brown women, were considered as the exposure group and white race/color women were considered a reference group.

Two review authors independently analyzed the entire process of study selection, analyzing titles and abstracts. The articles were read in full, following the established inclusion and exclusion criteria.

Data from each eligible article for the study were extracted and organized into a summary table which contained the following points: authors and date of publication, study outline, and association measures. The articles were evaluated for methodological quality using the Newcastle-Ottawa Scale (NOS) instrument, which evaluates the quality of cohort, cross-sectional and case-control studies for the detection of bias^17^. Studies with poor methodological quality were excluded. The quality of the studies was based on the classification of the articles in the following categories: bad, regular, good and excellent.

The studies were analyzed regarding the methodological quality in the following questions: regarding the selection of the studies, in which the representativity of the sample was observed; the exposure and the outcome of interest; the comparative power of the findings; and the effect through the evaluation of results. We obtained the following classification of the articles: three regular, 10 good and four excellent.

The collected data were expressed in absolute numbers and inserted in contingency tables (2x2) to calculate the measure of association. We selected studies that reported only primary data. Descriptive and meta-analytical analyzes of the data were performed. The relative risk (RR) and the 95% confidence interval were used as measures of effect.

To verify the heterogeneity and the consistency of the studies, Cochran’s Q test and I^2^ was used. The random model and the Egger regression model were used, generating the forest plot graph.

The results of the occurrence of the PMT associated with race/skin color were meta-analyzed by means of the statistical program Stata.

After searching the BVS using the descriptors association, 1,163 results were found, of which 372 were published as of 2010. The search in MedLine and PubMed using the same word association found 1,907 results, 554 published as of 2010 (Table 1).

**Table 1:** Distribution of the search results of the articles on race/skin color and prematurity according to the associations of the descriptors and the databases.

Results of the association of descriptors	PubMed		BVS	
	
	General	Study period (2011–2014)	General	Study period (2010–2014)
“Race or ethnic group” AND “infant premature”	1,480	481	661	166
“race” AND “obstetric labor premature”	421	26	105	18
“ethnicity and health” AND “infant premature”	3	2	328	173
“ethnicity and health” AND “obstetric labor premature”	3	3	69	15

Total	1,907	554	1,163	372

BVS: Virtual Health Library

**Figure 1:**
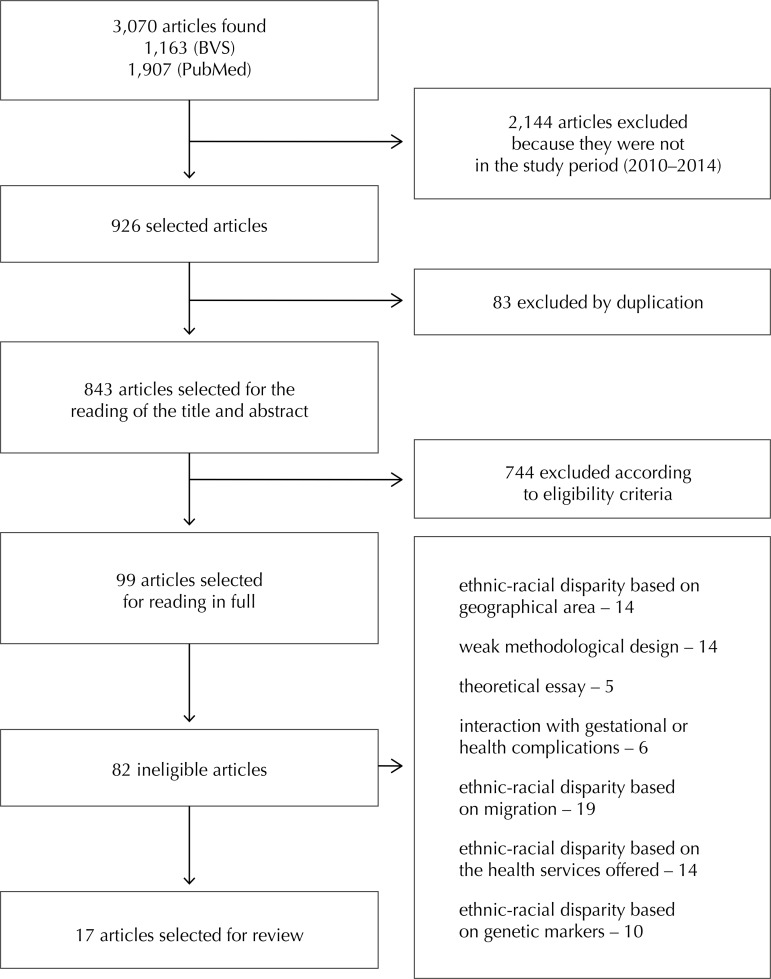
Flowchart for the selection of observational studies evaluating race/skin color associated with prematurity.

We identified 3,070 records in the databases, of which 926 articles related to the period from 2010 to 2014. Of this total, 83 articles were excluded by duplication (Figure 1). An exploratory reading was carried out with the objective of obtaining an overview of the 926 articles, with a reading of the titles and abstracts to verify what would be included in the study.

A total of 843 articles were excluded because they did not meet the inclusion criteria, resulting in 99 articles for reading in full. After the analysis based on readability criteria, 17 articles were included; a thorough reading and extraction of the data was done. The 17 published full texts adopted as a criterion for determining race/color self-classification, based on the color and physical characteristics of the participants. Of these, seven were retrospective cohort studies, nine were cross-sectional studies and one was a case-control study.

The reasons for the exclusion of the articles were: ethnic-racial disparity based on geographical area (n = 14), weak methodological design (n = 14), theoretical essay (n = 5), interaction with gestational or health complications (n = 6), ethnic-racial disparity based on migration (n = 19), ethnic-racial disparity based on health services offered (n = 14), and ethnic-racial disparity based on genetic markers (n = 10).

## RESULTS

Most articles (35.3%) were published in 2013 and all articles (100%) were conducted in the United States. For the classification of gestational age, the date of the last menstruation (DLM) was applied as the defining criterion in all studies and the non-black race as the reference category. Except for one article^26^, the selected studies reported an increased risk or adjusted odds of preterm birth within the black ethnic/racial group investigated when compared to non-black (Table 2).

**Table 2:** Synthesis of articles assessing the association between race/skin color and prematurity, 2010 to 2014.

Author/Year	Place of study	Type of study	Sample (n)	Maternal age	Prematurity (%)	Incidence among black women	Incidence among non-black women	Adjusted measure of association	Quality of the article
Almeida et al.^27^ (2014)	New York/USA	Cross-sectional	4,443	80% under 35 years	8.0	14.6	5.4	OR = 3.01	Good
Castrillio et al.^28^ (2014)	Chicago/USA	Cross-sectional	267,303	Up to 35 years	5.3	19.8	9.0	RR = 1.2	Excellent
Collins Jr et al.^29^ (2013)	Chicago/USA	Cross-sectional	267,303	60% (20 to 29 years)	7.2	15.1	6.7	OR = 1.9	Good
Flores et al.^30^ (2012)	Chicago/USA	Cross-sectional	196,617	86% (20 to 34 years)	14.0	9.0	8.1	OR = 1.04	Good
Hwang et al.^31^ (2013)	Washington and Montana/USA	Retrospective cohort	24,648	< 18 years	9.6	11.0	8.0	OR = 1.34	Good
Shempf et al.^32^ (2011)	North Carolina/USA	Cross-sectional	31,489	50% (25 to 34 years)	7.4	13.1	6.9	RR = 3.0	Excellent
Shaw et al.^33^ (2010)	USA	Retrospective cohort	1,223,751	20 to 35 years	12.5	15.6	10.17	OR = 1.37	Good
Shaw et al.^39^ (2013)	USA	Retrospective cohort	2,646,176	20 to 29 years	9.9	15.6	8.5	OR = 1.09	Good
Sullivan et al.^34^ (2012)	Texas/USA	Cross-sectional	369,839	≥ 18 years	13.7	18.4	12.3	OR = 0.48	Excellent
Whitehead; Helms^40^ (2010)	New York/USA	Cross-sectional	343,988	18 to 34 years	7.2	7.3	7.8	RR = 0.95	Good
Xiong; Pridjian; Dickey^41^ (2013)	USA	Retrospective cohort	50,377	> 70% (30 to 39 years)	13.7	24.0	12.9	OR = 2.1	Regular
Zhang et al.^35^ (2013)	USA	Cross-sectional	1,472,912	> 50% (18 to 24 years)	5.7	10.0	7.2	OR = 1.34	Regular
Mohamed et al.^36^ (2012)	USA	Retrospective cohort	17,338	25 to 35 years	10.1	22.1	12.8	OR = 1.61	Good
Coley; Aronson^42^ (2013)	North Carolina/USA	Cross-sectional	10,515	17 to 19 years	8.4	11.0	9.2	-	Regular
Jongh et al.^38^ (2014)	USA	Retrospective cohort	11,711	20 to 34 years	3.9	13.5	8.8	OR = 1.36	Good
Fujimoto et al.^37^ (2010)	USA	Retrospective cohort	139,027	35 to 39 years	15.2	21.0	14.8	OR = 1.79	Excellent
Torloni et al.^26^ (2012)	Tennessee/USA	Case control	1,762	20 to 34 years	8.3	21.7	27.6	OR = 1.29	Good

**Figure 2:**
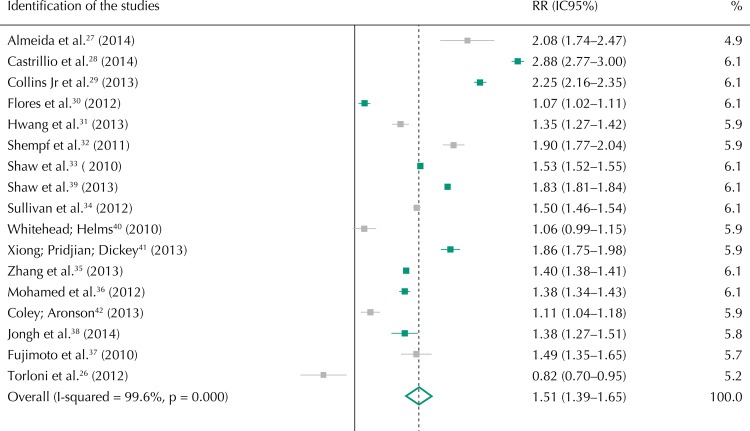
Forest plot of observational studies on race/color association and prematurity.

Note: Values are obtained by means of random effects analysis.

The 17 studies were grouped comparing the risk of the occurrence of preterm birth among black and non-black women. Articles^27^ ^-^ ^32^ showed a positive association between race/skin color and the occurrence of PMT. Black women presented a 51.0% higher risk of preterm birth compared to non-black women (RR = 1.51, 95%CI 1.39–1.65). The results of the inconsistency test showed high heterogeneity among the analyzed studies (99.6%; p = 0.00). Thus, the random effects model was used to calculate the synthesis measurement (Figure 2).

**Figure 3:**
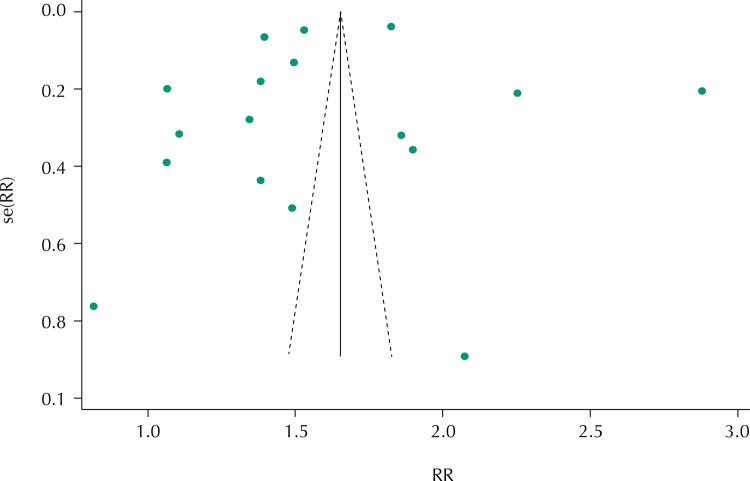
Funnel graph of the association between race/skin color and prematurity.

The results showed great variability among the studies, which denoted the presence of publication bias (Figure 3). Five larger studies appeared at the top of the chart, 10 studies with mean samples located in the centermost part to the left of the chart and two studies at the bottom because they had smaller samples. All studies presented outside the graph slope.

## DISCUSSION

The results of the present meta-analysis confirm the association between race/skin color and the occurrence of PMT: black women were 1.5 times more likely to have preterm birth when compared to non-black women. This result supports the hypothesis that skin color/race is a risk factor for prematurity and corroborates the findings of previous publications of observational studies and systematic reviews^7^ ^,^ ^16^ ^,^ ^33^.

The association between race/skin color and PMT does not have its mechanism fully clarified from the perspective of social determination, taken as the theoretical framework in this study. However, the literature refers to maternal factors that interfere with prematurity such as height and weight of the mother, parity, and complications during pregnancy, adolescent mothers (especially if < 15 years). Also mentioned are the behavioral factors that influence PMT, including smoking, alcohol and drug use during pregnancy and prenatal care. The socioeconomic condition, measured by family income, having housing, occupation, education, the type of maternal work and solitary motherhood also influences PMT. All of these risk factors intersect for premature birth and are more strongly present in the life of black women because of the disadvantage that they are destined for by society. Black women generally have worse socioeconomic status and worse condition of having good nutrition. Moreover, they are exposed to discrimination because of their ethnic-racial identity and this psychological stress can also lead them to have preterm children^1,34–36^.

It is believed that the interaction between genetic and environmental factors influences the race/skin color as one of the causes for the occurrence of PMT^37^. In this sense, ethnic-racial inequalities can be seen in access to health services, socioeconomic opportunities, and genetic factors. It thus constitutes an unequal situation faced by black women.

Included studies were scored as good or excellent after quality assessment. A large variation in the sample size of the included studies can be observed. The study by Torloni et al.^26^ presented 1,762 women and the study by Shaw et al.^38^ presented 2,646,176 women. However, the sample size had minor impact on the generalization of the results.

Only in the study of Hwang et al.^31^ the majority of the participants were below 18 years old. As there was an adjustment of this covariate in this study, it is believed that it did not influence the results of this meta-analysis. Likewise, variables that could be associated with the occurrence of PMT, such as socioeconomic and behavioral factors, also did not influence the meta-analytic measure in the present study, despite the high heterogeneity identified. This indicates the variation between the results of the analyzed studies.

For all included studies, information on gestational age was obtained from a hospital or health service registry and DLM was adopted as a defining criterion for gestational age. This procedure reduced the possibility of memory bias.

The included studies showed a great variation in the occurrence of PMT. The rates of prematurity in black women varied from 7.3%^39^ to 24.0%^40^ and this variation is even greater among non-black women, which was 6.7^41^ to 27.6^42^. For the adjusted measure of the association between race/color and PMT, the highest prevalence of PMT was 8.0%^27^. The authors showed that there were significant differences by race/color in all sociodemographic, behavioral and medical variables, except for a previous history of preterm birth.

Black women were more likely to have a preterm birth than non-black women. Comparable results were found in the UK study, in which black women were 1.5 times more likely to have preterm births^19^. These findings express the difficulty of access to health services by women and children of different ethnic groups. This may explain the differences found in PMT occurrence levels due to the absence of prevention of avoidable risks from comprehensive care.

Regarding the sample size, the study by Torloni et al.^26^ presented a smaller number of participants, which can be explained by the method used, which is case-control. The study presented a prevalence of 35.4% for the occurrence of preterm birth in the total study population. However, there was a higher prevalence among non-black women when compared to black women, which differs from most of the selected studies. One of the limitations of that study was the small number of participants, which may have made it difficult to analyze the effects of PMT in women of different ethnic origins.

The study by Xiong et al.^41^ showed the occurrence of preterm birth in black women of 4.8 (95%CI 4.1–5.7), when compared to white women and presented a prevalence of 13.7% for the study. One of the main limitations of the study was in relation to race/color information. About 35.0% were missing and in relation to the control of confounding factors, since there was a frequent lack of information on obstetric data and on pre-existing clinical conditions. However, the study has as its strong point the sample size, sufficient to analyze the association between race/color and prematurity.

For most studies, information on birth weight was obtained from a hospital registry, reducing the possibility of memory bias. However, the number of articles included in this meta-analysis was small in relation to the number identified in the systematic literature review. As a result, there is a potential selection bias.

Most of the studies included in this meta-analysis were cross-sectional, limiting the ability to distinguish temporal relationships underlying the association between race/skin color and the occurrence of PMT. However, this is a limitation of the studies currently published in this field. As this is a meta-analysis of observational studies, this study faced the inherent challenge of summarizing the results of the studies with different epidemiological delineations.

The results of the present meta-analysis evidenced the association between race/skin color and PMT. Black women are 1.51 times more likely to have a preterm birth than white women. This finding can support decision making and provide elements that support efforts to achieve equity in health.

However, skin color/race affiliation and PMT present complex aspects social, behavioral and biological. Future studies may investigate an association between race/skin color and PMT, in order to consider these aspects, mainly to black ones, due to your unfavorable social and economic conditions. This situation is exacerbated by the treatment that society gives to certain groups when considering their ethnic-racial affiliation.
